# Deep Learning Algorithm for Differentiating Patients with a Healthy Liver from Patients with Liver Lesions Based on MR Images

**DOI:** 10.3390/cancers15123142

**Published:** 2023-06-11

**Authors:** Maciej Skwirczyński, Zbisław Tabor, Julia Lasek, Zofia Schneider, Sebastian Gibała, Iwona Kucybała, Andrzej Urbanik, Rafał Obuchowicz

**Affiliations:** 1 Faculty of Mathematics and Computer Science, Jagiellonian University, 30-348 Krakow, Poland; 2 Faculty of Electrical Engineering, Automatics, Computer Science, and Biomedical Engineering, AGH University of Science and Technology, 30-059 Krakow, Poland; 3 Faculty of Geology, Geophysics, and Environmental Protection, AGH University of Science and Technology, 30-059 Krakow, Poland; 4 Ultragen Medical Center, 31-572 Krakow, Poland; 5 Department of Diagnostic Imaging, Jagiellonian University Medical College, 31-501 Krakow, Poland

**Keywords:** liver tumor segmentation, classification, deep learning, focal lesions, hepatocellular carcinoma, adenoma, focal nodular hyperplasia, magnetic resonance imaging (MRI)

## Abstract

**Simple Summary:**

We propose an automated system that handles numerous problems encountered when diagnosing the presence of numerous types of lesions in the liver based on multiparametric magnetic resonance (MR) images. Using a properly built and processed dataset and deep-learning segmentation algorithms, we devised a method for screening MR images for the presence of focal lesions in the liver.

**Abstract:**

The problems in diagnosing the state of a vital organ such as the liver are complex and remain unresolved. These problems are underscored by frequently published studies on this issue. At the same time, demand for imaging diagnostics, preferably using a method that can detect the disease at the earliest possible stage, is constantly increasing. In this paper, we present liver diseases in the context of diagnosis, diagnostic problems, and possible elimination. We discuss the dataset and methods and present the stages of the pipeline we developed, leading to multiclass segmentation of the liver in multiparametric MR image into lesions and normal tissue. Finally, based on the processing results, each case is classified as either a healthy liver or a liver with lesions. For the training set, the AUC ROC is 0.925 (standard error 0.013 and a *p*-value less than 0.001), and for the test set, the AUC ROC is 0.852 (standard error 0.039 and a *p*-value less than 0.001). Further refinements to the proposed pipeline are also discussed. The proposed approach could be used in the detection of focal lesions in the liver and the description of liver tumors. Practical application of the developed multi-class segmentation method represents a key step toward standardizing the medical evaluation of focal lesions in the liver.

## 1. Introduction

The liver is a vital organ unique for metabolism, in which numerous biochemical pathways are present [1]. Due to the liver’s complexity and unique functional role, liver health and preservation of the liver parenchyma represent major causes of concern. The liver, as the only place where certain processes occur (such as detoxification), is vulnerable to damage caused by various toxins, with alcohol being the most prevalent among the population [2,3]. Prolonged exposure to toxic substances leads to cirrhosis with a high incidence of hepatocellular carcinoma [4]. Hepatocellular carcinoma is the most common primary liver cancer [5]. The probability of developing HCC is more than 20% in the cirrhotic liver, and outcome of the tumor is fatal [6]. Due to its involvement in the hormonal pathways, the liver is also subject to the influence of the both endogenous and exogenous hormones, which are related to the formation of lesions such as FNH [7,8]. Hormonal simulation can also induce the dimensional growth of hemangiomas [9]. Finally, the liver’s anatomy itself, due to its many tubular structures filled with fluids, promotes the formation of cysts. Consequently, benign cysts are the most frequent lesions [10]. The importance of the liver itself and probability of different lesions being formed makes the liver a point of interest in evaluations using diagnostic imaging procedures. Magnetic resonance is the most important modality in the diagnosis of the most common primary form of liver cancer: hepatocellular carcinoma. The diagnostic accuracy of MRI in the detection of liver tumors is high compared to that of tumors in other organs [11,12,13]. According to international guidelines, MRI is the first step of the diagnostic process, and histopathological analysis is required for liver nodules with an atypical imaging presentation [14,15]. However, a liver biopsy is not always feasible, especially in the case of very small liver nodules; furthermore, this procedure is not free from risks and is limited by an elevated rate of false negatives (about 30%) [16]. Therefore, the implementation of radiomics and machine learning software in clinical practice appears to be a promising and interesting new field of investigation.

Sensitivity and specificity for MRI-supported diagnosis are over 90% in hemangioma, cyst, and FNH detection [17,18,19,20]. With this high detection sensitivity, MRI outperforms CT in the detection of lesions with diameters less than 2 cm [21]. Notably, MRI is also advantageous in the detection and characterization of intracellular compounds, which may indicate the state of disease or even level of advancement of morbidity. The use of hepatobiliary contrast agents and the introduction of diffusion-weighted imaging sequences in the protocol supported the recognition of premalignant lesions helps to assess risk in atypical or indeterminate nodules [22,23]. Importantly, MR requires no radiation exposure, which is especially important in abdominal CT, for which the dose is relatively large, ranging from 15 to 24 mSv and corresponding to a dose equivalent to 220 to 350 single chest X-rays. This exposure may influence lifetime attributable cancer risk [24]. Although MRI efficiency is reported as remarkably high, it must be noted that those numbers represent the best human experience in the detection of lesions reported in diagnostic processes. Human perception of the patterns of textures present in the analyzed image plays a key role in the diagnostic process [25]. Detection relies on differences in the lesion textures compared to the background. Recognition is a process based on a comparison of the perceived textures against the “gold standard” lesion attributes as specified in, e.g., atlas recommendations. This process is subjective and prone to errors and intra-reader variability, which makes computer-based supporting systems highly anticipated by the medical community.

In the past years, an enormous increase in the number of medical imaging examinations performed has been observed [24]. This trend is connected to an increase in demand for radiological opinions in clinical decision-making processes [26]. Increasing the number of imaging exams introduces large amounts of data, which cannot be analyzed in the time expected by patients. Therefore, the concept of automated image analysis is growing in popularity as a promising approach for obtaining results faster and more effectively in order to meet clinical needs [27]. Among the problems related to the automated analysis of medical images, the task of screening for the presence of cancer mass is emerging and continues to gain popularity, especially in the fields of lung cancer and brain lesions but also in liver cancer detection [28,29,30]. The use of automated analysis methods such as radiomics and machine learning improves cancer detection in early stages, which is connected to an increased survival rate [31]. This increase is often connected to the detection of incipient cancer, which in the worst case scenario, could potentially be overlooked by a human [32].

Recently, studies have presented many automatic and semi-automatic methods based on classical methods of image analysis and deep learning (DL) techniques, aimed at creating systems for the reliable diagnosis and detection of liver tumors. The proposed approaches are mostly applied to CT images of livers and based on available public liver CT databases. Given that the liver can undergo various pathologies such as hepatic fat, iron deposits, fibrosis, and tumors, which can modify the liver’s density and signal intensity or simply distort its shape, existing methods suffer from inaccuracies. Another important aspect is the location of the liver, whose immediate surroundings, due to having a similar intensity, add to the difficulty of corresponding problems. Liver segmentation is one of the key steps in various clinical applications such as tumor detection and is often preceded by the preprocessing required to achieve high accuracy in later stages. Preprocessing can include applying windowing to the raw image [33]; histogram equalization, the most widely used being contrast limited adaptive histogram equalization [34]; data normalization [35]; image denoising, e.g., median filter [36]; MR bias field correction, e.g., N4 bias correction [37]; or the joint removal of MRI bias [38].

MR images provide much better differentiation between healthy tissues and lesions. Nevertheless, automated methods for the analysis of these images are not common, especially when it comes to the analysis of multiparametric MR images of the liver. In light of these facts, in our study, we analyze T1 (pre- and post-contrast), T2 TSE, and DWI. We propose a DL-based processing pipeline aimed at screening MR liver examinations for the presence of lesions. Because multiparametric MR liver databases are not available in the public domain, as a part of this study, an imaging database was collected, which includes both MR abdominal examinations and reference manual segmentation of the liver and lesions.

## 2. Materials and Methods

### 2.1. Materials

The study protocol was developed based on the guidelines of the Helsinki Declaration and the Declaration of Good Clinical Practice. All images were anonymized prior to processing to ensure the security of personal data. In addition, written consent from the Local Ethics Committee was obtained to conduct this study 1072.6120.22.22 (23 February 2022).

**Figure 1:**
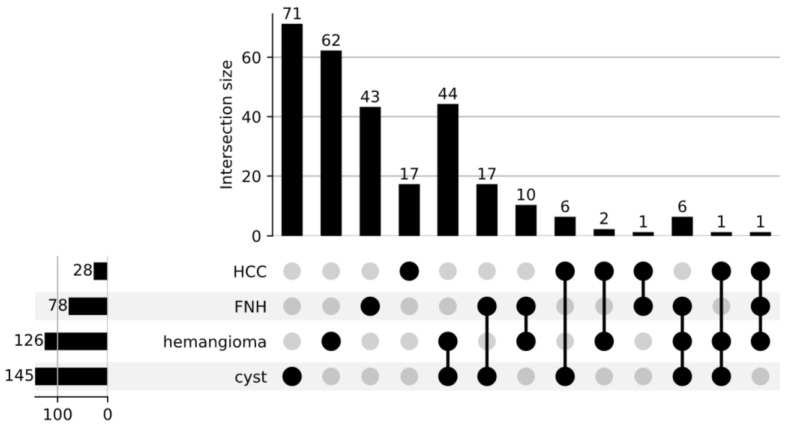
Distribution of the focal lesions in the examined set of approved images.

Images were selected in the Hospital Data Reporting System—Hospital Information System (HIS). To select the group of interest, 6500 records were reviewed by data scientists and the radiology team. From this dataset, 1520 records were selected for further evaluation and examined in the hospital PACS for the presence of lesions (hepatocellular carcinoma, hemangioma, liver cysts, and focal nodular hyperplasia). The control group, with no lesions confirmed, was also selected from the database. In total, 500 records that met the quality criteria (free of artefacts and completeness of the desired sequences) were exported for further analyses. Meticulous scanning and verification of the lesions revealed 145 cystic lesions (C), 126 hemangiomas (HM), 28 hepatocellular carcinomas (HCC), and 78 focal nodular hyperplasia (FNH) cases. For some patients, more than one type of lesion was present in the liver. The distribution of particular lesions is given in Figure 1.

In this study, we used data from the selected 500 patients scanned in a 1.5 T MR system. MRI images were acquired using the standard diagnostic protocol for patients referred for an abdominal evaluation in MRI. DWI, single-shot echo-planar imaging, EPI, T2 turbo-spin echo, T1 turbo-spin echo, post-contrast early (T1 + C), and T1 turbo-spin echo post-contrast (non-hepatospecific) delayed (T1 Delayed) were used in in this study. Diffusion weighted sequences (EPI) were produced with 50-400-800 B using 2, 4, and 8 averages, with three diffusion weightings. In all applied sequences, the signal/noise ratios were equal to 1. The protocol outline of sequences used for the evaluation is presented in Table 1.

**Figure 2:**
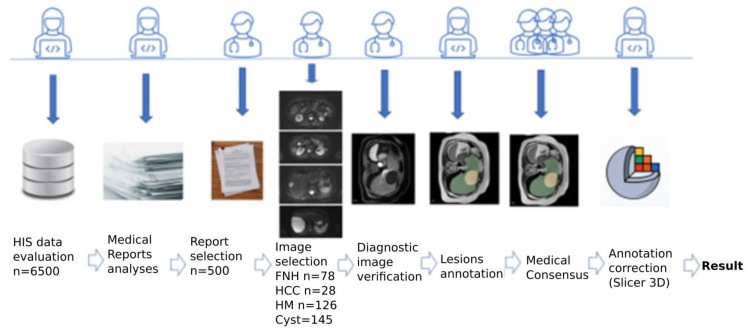
Workflow for collecting from the image database (designed as a “Result” in the figure) used to develop deep-learning algorithms.

The reference manual segmentation of the liver and lesions was prepared with the 3D Slicer Imaging Computing Platform (https://www.slicer.org/, accessed on 1 February 2023). For each patient, the liver was segmented in each MR sequence. For each lesion, the lesion’s presence was confirmed, and its outlines were drawn for each slice in which the lesion was visible. Each lesion was outlined for a single MR sequence where the lesion was most prominent, as determined using a visual assessment. The liver and lesion outlines were prepared by two members of the research team. The initial outlines were then verified by a group of three radiologists (with 40, 15, and 6 years of experience) who applied an intrareader multistep consensus procedure to arrive at the final outlines. Agreements over 92% were accepted for further image analysis. Lower agreement resulted in corrections of the initial outlines. The workflow is presented in Figure 2.

### 2.2. Methods

The dataset of 500 MR examinations was randomly split into training and testing portions. Stratified sampling was applied to the training and test sets when selecting examinations: approximately 20% of examinations were selected for the test set from each group of examinations (healthy, with HM, with HCC, with cyst, with cyst and FNH, etc.). The training set consisted of 405 MR examinations, including 178 examinations in which no lesions in the liver were visible and 227 examinations in which lesions were present. The test set consisted of 95 MR examinations, including 43 examinations in which lesions in the liver were not visible and 52 examinations in which changes were present.

To complete the task of patient classification, we devised a pipeline consisting of the steps described below. Firstly, the four diagnostic images, denoted T2, T1 + C, T1 Delayed, and DWI in the following, were loaded from the corresponding DICOM sequences and entered into a spatial mesh with unified spatial resolution based on linear interpolation and information stored in appropriate DICOM tags (pixel size and slice thickness equal to inter-slice distance).

In the next step, the liver in each of the four images was segmented. For this process, we used U-Net, as implemented in nnU-Net framework [39]. The nnU-Net implementation of U-Net differs slightly from that of the vanilla U-Net [40]. Most notably, in nnU-Net, stride convolutions were used instead of max-pooling, and Leaky ReLU was used instead of plain ReLU. There was also a difference in data preparation. The authors of nnU-Net implemented a universal framework, which means that this framework can be used for very different datasets, often with better results than neural networks solutions aimed at operating on only one concrete dataset. Network topology details are also established based on datasets. This process allows for the appropriate aggregation of spatial information and is also influenced by hardware parameters, such as GPU memory. To account for these factors, we started with the initial patch size and verified that the size would fit within the GPU memory. If so, the batch size was increased; if not, the patch size was decreased. In addition to input patch size and number, pooling operations per axis were set, which also implicitly set the number of convolutional layers [41]. For the above-mentioned neural network, we chose training in a fivefold cross-validation scheme, where the training set was randomly divided into five parts. Four of these parts were used as the actual training set and one as the validation set. The validation set was selected in five possible ways by running five training sessions and obtaining five models of liver segmentation. These five models were used in the prediction stage by averaging their responses.

Data augmentation was applied during the dataset preparation stage and was implemented using the Batchgenerators framework [42] with the following augmentations [39] (the value of the probability of applying each augmentation is given in parenthesis): Spatial: rotation, scaling (0.4)—in the case of isotropic patches, the angles of rotation around each axis, given in degrees, were sampled from U (−30, 30), and for anisotropic patches, from U (−180, 180); the scaling factor was sampled from U (0.7, 1.4). Gaussian noise (0.15), if applied, was added to each voxel independently with the variance of the noise drawn from U (0, 0.1). For Gaussian blur (0.2), the width of the Gaussian kernel, expressed in voxels, was taken from U (0.5, 1.5), and the blur (0.5) was applied to each modality independently. For brightness (0.15), the intensity of each voxel was multiplied by the value drawn from U (0.7, 1.3). For contrast (0.15), the intensity of each voxel was multiplied by the value drawn from U (0.7, 1.3); the latter was subsequently trimmed to the original range. A simulation of low resolution (0.25) was achieved using nearest neighbor interpolation to downsample the modalities by a U (1, 2) factor and then resample the modalities back to their original size using cubic interpolation. The gamma intensities (0.15, and the voxel intensities were inverted beforehand) were scaled to a factor [0, 1] of their respective range of values, a nonlinear transformation of the intensities per voxel was applied, and then the intensities were scaled back to their original range of values. Mirroring (0.5) was performed with a given probability along all axes. After liver segmentation, we removed all but the largest connected components. This process was found to be beneficial for our task.

Then, we performed registration between T2 and the remaining diagnostic images. The registration step was necessary because, typically, the effects of a patient’s movements between acquisition of the four sequences were clearly visible. We first found three transformations between the liver segmentations in T2 images, which we set as fixed images. These transformations were, respectively, T1 + C, T1 Delayed, and DWI liver segmentations and were set as moving images. We used a method that allows for two-stage registration, where each stage is characterized by different transforms. At the beginning of the first stage, we set a similarity 3D transform with rotation, uniform scaling around a fixed center, and translation. To ascertain the agreement between the two registered images, we used negative normalized cross correlation with the sampling strategy set to random (sample image voxels with replacements using a uniform distribution) and the sampling percentage set to 1%. At this stage, we used a linear interpolator and regular step gradient descent optimizer. The second stage was implemented in an analogous way. We changed the transform to B-spline and the optimizer to Limited memory Broyden, Fletcher, Goldfarb, Shanno, and Bound Constrained (LBFGSB) [43,44]. Subsequently, we applied the found transformations to the MR images obtained by combining all sequences under one spatial mesh. After the registration step, we cropped the images to the region of liver segmentation.

We then fed the data into nnU-Net, which was trained in the same way as before, only this time for simultaneous liver and liver lesion segmentations. The output returned multiclass segmentation of the liver and lesions.

**Figure 3:**
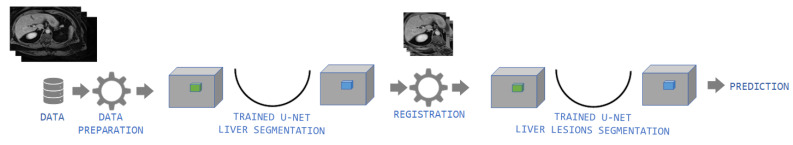
MR image processing workflow.

The ultimate screening task was solved based on the results of the multiclass segmentation. Whenever the segmentation indicated that the number of voxels labeled as being contained within a lesion was larger than a certain decision threshold, the MR examination was labeled as containing a lesion. ROC curves were then obtained by varying the decision threshold in a range from 0 to 100,000 voxels. Additionally, the liver and lesion segmentation quality were measured by means of two standard metrics: the Dice coefficient and robust Hausdorff distance (with tolerance set to 0.99). Computation of the segmentation quality metrics was accomplished using the surface–distance library (https://github.com/deepmind/surface-distance, accessed on 1 February 2023). The processing workflow is shown in Figure 3.

## 3. Results

**Figure 4:**
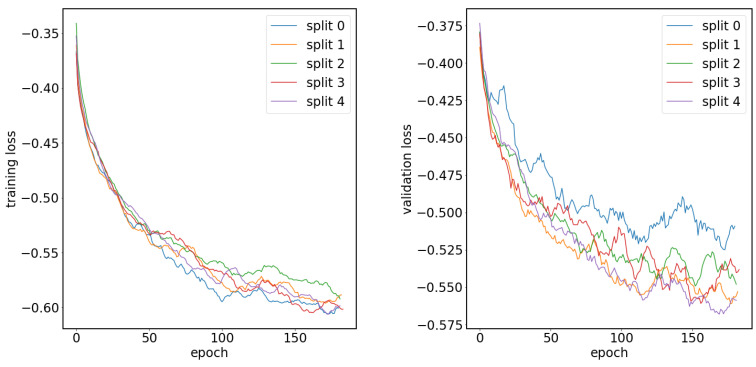
Plots of the loss function for the task of liver segmentation in T2 images for the training sets (left) and validation sets (right).

**Figure 5:**
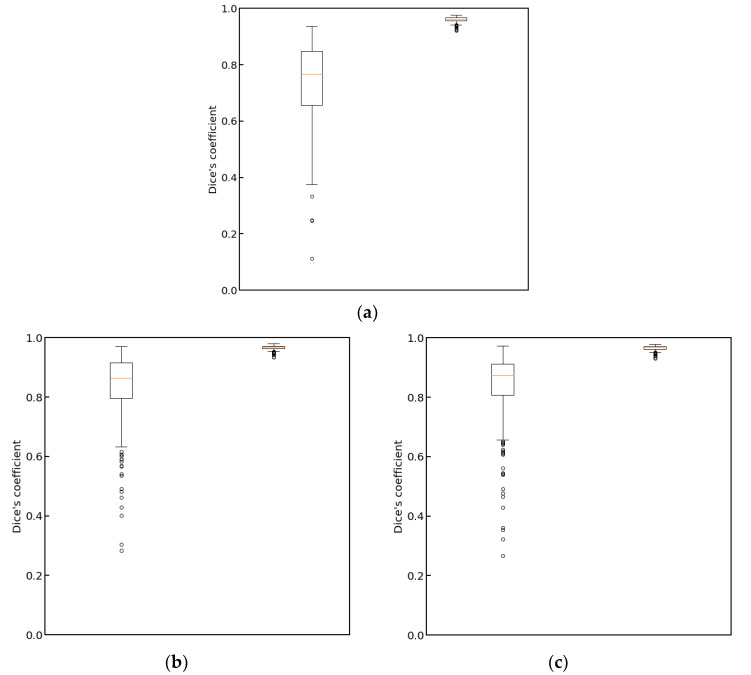
The results of registering moving images: (**a**) DWI, (**b**) T1 + C, and (**c**) T1 delayed against the fixed image T2. In each plot, the Dice coefficients between liver segmentation in moving and fixed images are shown before registration (left box plots) and after registration (right box plots).

**Figure 6:**
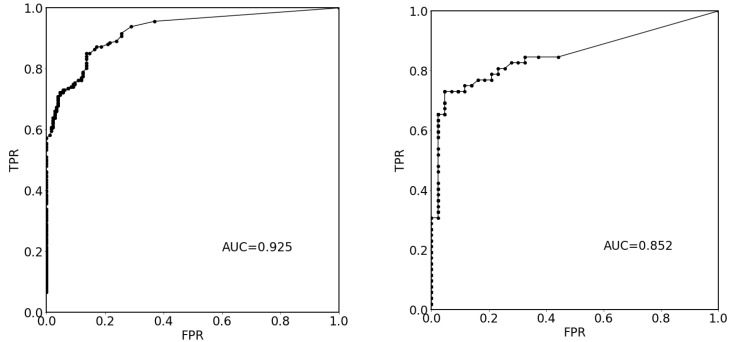
ROC curve for the “healthy liver/liver with a lesion present” classification task. On the left, the figure presents results for the training set. Here, the area under the ROC (AUC ROC) is equal to 0.925 (standard error 0.013, *p*-value less than 0.001). On the right, the results for the test set are presented. Here, the AUC ROC is equal to 0.852 (standard error 0.039, *p*-value less than 0.001). TPR: true positive rate; FPR: false positive rate.

**Figure 7:**
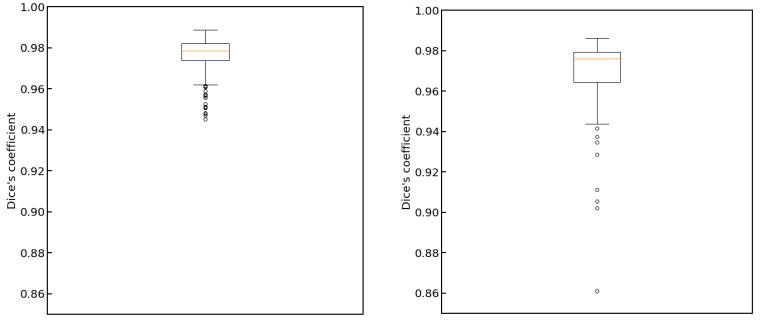
Box plots for the liver segmentation task. On the left, the results for the training set are presented. On the right, the results for the test set are presented.

**Figure 8:**
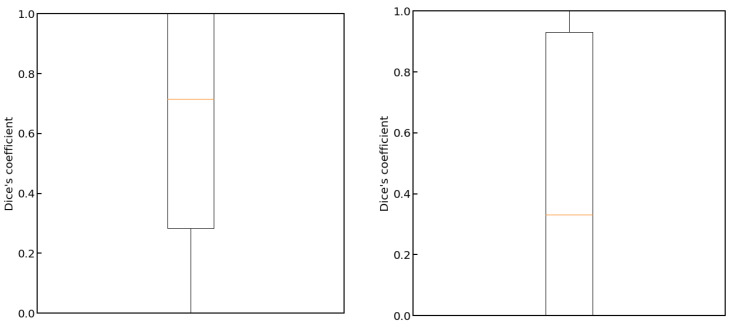
Box plots for the liver lesion segmentation task. On the left, the results for the training set are presented. On the right, the results for the test set are presented.

**Figure 9:**
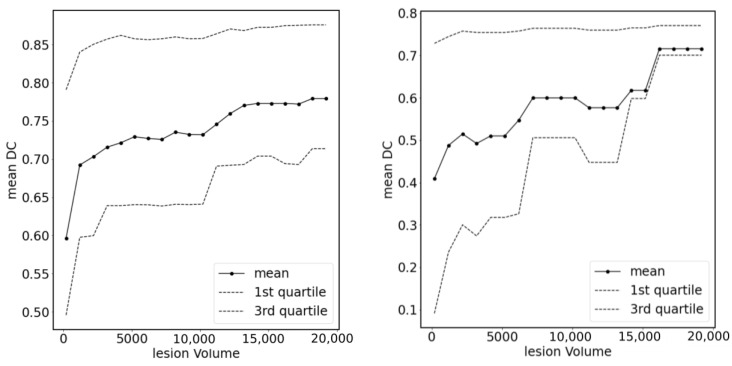
Accuracy of hepatic lesion segmentation measured by the Dice coefficient, presented according to lesion volume (measured by number of voxels). On the left, the results for the training set are presented. On the right, the results for the test set are presented.

In Figure 4, we show plots of the loss function for the task of liver segmentation in T2 images. The U-Net models were trained until a plateau of the loss function was reached for a validation set. During training, the best models for the validation set were saved and subsequently used for predictions. The training and validation curves for other segmentation tasks looked similar to the examples shown in Figure 4. In Figure 5, we show the results of registration between T2 (fixed) images and the remaining diagnostic images. Clearly, the effect of patient movement was very prominent, as demonstrated by relatively low values of the Dice coefficient before registration. Registration compensated for patient movement to some extent, as the Dice coefficient after registration was around 96%. Figure 6 shows the ROC curves for the training and test sets in the screening task. The ROC AUC value was found to be substantially higher for the training set than for the test set. Given the number of true positive and true negative cases in the training and test sets, the ROC AUC values equal to 0.925 (standard error 0.013) and 0.852 (standard error 0.039) for the training and test sets, respectively, were statistically significant at a *p*-value less than 0.001. After fixing specificity (SPE) at 0.79 for the test set, the sensitivity (SEN) of detection for patients with liver lesions was equal to 0.79. For the test set and decision threshold equal to 100 voxels, the SEN, SPE, and accuracy (ACC) of classification were equal to 0.79, and the positive predictive value (PPV) was equal to 0.82. The corresponding values for the training set were 0.85 and 0.88. For the test set and the decision threshold equal to 300 voxels, the ACC was equal to 0.83, SEN was equal to 0.73, SPE was equal to 0.95, and PPV was equal to 0.95. The respective values for the training set were 0.82, 0.75, 0.91, and 0.91. Figure 7 shows box plots for the liver segmentation task. The values of the Dice coefficient were found to be quite high at about 0.98. In contrast, the task of lesion segmentation is not solved equally well, as demonstrated by the box plots for the liver lesion segmentation task (Figure 8). Figure 9 demonstrates that the accuracy of hepatic lesion segmentation, measured by the Dice coefficient, was strongly dependent on the lesion volume measured by the number of voxels, with the segmentation of larger lesions being more accurate. These conclusions regarding segmentation quality were further supported by the values of robust Hausdorff Distance (rHD). For the task of liver segmentation, the median rHD in the training set was equal to 1.0 voxels, standard deviation was equal to 2.5 voxels, and the 95% quantile was equal to 4.0 voxels. The respective values for the test set were 1.4, 5.5, and 15.2 voxels. For the task of lesion segmentation, the median rHD in the training set was equal to 23.2 voxels, the standard deviation was equal to 42.9 voxels, and the 95% quantile was equal to 193.5 voxels. The corresponding values for the test set were 25.9, 53.8, and 168.7 voxels.

## 4. Discussion

There are numerous possible lesions in the liver. These lesions can be aggressive in terms of growth and also represent a danger in replacing healthy parenchyma [45,46]. The impact of liver dysfunction on the health status of an individual is enormous, so there is a strong need for accurate diagnoses. Contemporary radiology with MR is the most effective modality used for the detection of soft tissue lesions. One of the most important limitations of this process is the ability of the human eye to find differences between lesions and the liver parenchyma in the most effective and repeatable way. The field training of imaging specialists, psychosomatic conditions, and overall light condition in the examination room are all recognized factors that can influence lesion detection [47,48]. The application of different sequences where various constituents of the lesions can be visualized aids in recognition of such lesions. However, this effect might be alleviated with lesion polymorphism [49]. Therefore, the first step of lesion recognition—a border setting between normal parenchyma and the lesion—is a segmentation task run based on training with multi-step verified man-made descriptions. The second task—recognition of the patterns related to lesions—relies on associated processes and requires significance experience from the reader, including the ability to engage in comparative interpretation [50]. Both steps are part of daily practice in radiology, with special importance in oncology, where lesion dimensions and associated change are of particular importance in treatment assessment and further planning [51].

Deep learning methods are promising in the field of lesion detection and could help optimize the detection of liver lesions [52]. Some studies describing DL methodologies explored combined liver segmentation and liver tumors, but many focused only on liver segmentation. The 3D Deep Supervised Network described by Dou et al. [53] is based on a fully convolutional network architecture. The presented framework was evaluated on the MICCAI SLiver07 CT data ASD set. For this architecture, the total inference time for a single subject was 5 s for the 3D Deep Supervised Network and 87 s for conditional random fields, which corresponds to approximately one and a half minutes in total. Another algorithm using deep supervision was presented by Lv et al. [54]. The authors replaced the encoder’s standard convolution with a residual block and provided an atrous inception module to connect the encoder and decoder, allowing the model to obtain multi-scale features; the authors also incorporated a deep supervision mechanism into the decoder. In addition, the authors employed the Tversky loss function to balance segmented and nonsegmented regions and performed further refinements with a dense conditional random field. This method was validated on three public CT databases: LiTS17, 3DIRCADb, and SLiver07. As a drawback, the authors noted that the large amount of 3D network parameters hindered and slowed the training process. The authors also noted the susceptibility of the method to errors when considering livers with borderline tumors. Additionally, since this work was aimed at segmenting the liver, it may not be suitable for segmenting liver tumors. A two-step method was presented by Lu et al. [55]. In the first step, this method uses a 3D convolutional neural network to segment the liver. In the second step, segmentation accuracy is improved using cutting charts and maps. The model was tested on 40 CT volumes derived from the publicly available datasets Sliver07 and 3DIRCADb. The authors concluded that this method performs reasonably well compared to the manual method due to the latter’s long processing time and erroneous results. An evaluation of three different convolutional neural network (CNN) architectures was performed by Hoang et al. [56]. These architectures were a Fully Convolutional Network with Conditional Random Fields, Deep retinal image understanding, and the V-net model. The authors used contrast-enhanced images from the different datasets of LiTS, mayo, EMC_LD, EMC_NC_LD, which were collected from different medical centers. The results showed that all three CNNs achieved a mean Dice score of over 90% in liver segmentation with typical contrast-enhanced CT images of the liver, but these three architectures did not perform well for non-contrast-enhanced CT and low dose images. An automatic methods based on CNNs using 30 enhanced CT volumes were compared by Li et al. [57]. A Dice score of 80% was achieved in this study, demonstrating that the CNN methods performed better than the classical machine learning methods AdaBoost, Random Forests, and support vector machine. Li et al. [58] proposed a deep attention neural network including a high-resolution branch that can maintain input image resolution and thus preserve spatial details, as well as multiscale feature aggregation for cascading liver and tumor segmentation from CT images. This model achieved a Dice score of 76.3% for lesions and 96.0% for the liver when LiTS datasets were used for the evaluation.

For liver tumor segmentation in CT, the task is more difficult than liver segmentation. This process requires the patient to be injected with a contrast agent that enhances the contrast between the healthy liver and cancerous tissues of the liver. Even then, the variety of tumors makes this task difficult. However, the main reason segmentation of liver tumors is not frequently studied compared to whole liver segmentation is caused by the unavailability of public datasets with ground truth masks for liver tumors, which is due to the fact that manually annotating liver and liver tumor data is time consuming and prone to errors.

Magnetic resonance imaging has become the golden standard radiological modality for liver mass evaluation [59] due to the absence of ionizing radiation and good differentiation of morphological features of different tumor classes under this method [60]. With increasing clinical demand for MR-based diagnosis, there is a need to develop fast and comprehensive clinical protocols for liver mass detection and differentiation [61]. Proposed protocols consist of the sequences important for the detection of malignancies with less of a possible time expense [62]. Studies presenting different approaches showed the importance of T1-weighted pre- and post-contrast images but also T2-weighted images and DWI sequences, as summarized in the work of Brunsing [63]. These protocols called AMRI (Abbreviated MRI) protocols, enable the rejection of time consuming and costly abdominal procedures. The sensitivity and specificity of AMRI protocols were found to exceed 84% and 92%, respectively [64].

While the results presented in this study are promising, further research in this area is warranted to improve the quality of both screening and lesion segmentation. The differences between the results obtained for the training and test sets show that the trained models do not generalize very well. Since methods preventing overfitting (data augmentation) were intensively used during training, it can be concluded that in order to further improve the quality of the models, it is necessary to increase the training set. The relatively weak performance of the models in the task of lesion segmentation could also be a consequence of the heterogeneity of the dataset, which contains both very small and very large (e.g., 1/3 of the whole liver) lesions.

Further improvements are possible in the designed pipeline. The first step of liver segmentation in the diagnostic sequences was quite accurate (with a Dice coefficient above 0.98). While marginal improvements to liver segmentation are possible, it is unlikely that such improvements would improve the quality of the end-to-end process. The registration was also quite accurate (with a Dice coefficient around 0.96). However, because all four diagnostic sequences were used to segment the lesions, some small inaccuracies in the registration may lead to large inaccuracies in the segmentation of small lesions. For this reason, further research on high-quality inter-modality registration is required to improve end-to-end processing. Besides classic elastic registration, other methods were also tested (including ones based on deep learning, particularly the DeepReg framework; https://github.com/DeepRegNet/DeepReg, accessed on 1 February 2023), but no better results were obtained. Finally, after improving the inter-modality registration step and increasing the image database, keeping in mind the heterogeneity of the data, multi-class segmentation, which works well for large lesions, could be supplemented by object detector tools to detect small lesions. Deep-learning-based object detectors (MONAI library: https://monai.io/, accessed on 1 February 2023, nnDetection framework: https://github.com/MIC-DKFZ/nnDetection, accessed on 1 February 2023) were already applied to the current dataset, given the current registration algorithm, but the results were not sufficient to improve the end-to-end quality of the screening task. Today, there are tremendous efforts being undertaken to develop solutions based on radiomics analysis aimed at increasing the diagnostic utility of MR in the task of diagnosing liver lesions. Different approaches have been used to detect and classify liver lesions with promising results [65]. In the near future, it is expected that the expansion of computer-assisted classification systems developed in the field of liver malignancies will improve diagnostic accuracy to the level where making accurate diagnoses will be possible without expanding the process to invasive procedures. This measure will markedly increase patient comfort and safety.

The present study also has limitations, first of which is lack of adenomas and metastases in our database. Hemangiomas were rare in our material (over 30 cases) and were difficult to differentiate in the stage of manual segmentation preparation. Therefore, these hemangiomas were not ultimately used in the present study. The limited incidence of Hemangiomas is a consequence of the fact that these lesions are rare. Some studies reported a frequency of up to 20% in the general population [66], but in other studies, a frequency as low as 0.4% was found [67]. Metastases were not present in our material due to profile of the medical unit where the data were collected. Another limitation is that the analyses were based on four sequences only. The inclusion of more sequences may have improved the performance of the detection model. On the other hand, the inclusion of more sequences should be preceded by improving the inter-sequence registration algorithm. Otherwise, the benefits of using more data may be hindered by inaccuracies in the registration. The final limitation of our study is the size of the dataset, which, despite including 500 cases, was found to be too small based on a comparison of the results found for the training and test sets.

## 5. Conclusions

In this study, we designed a pipeline for liver screening. This method involves classifying examined patients into one of two groups: patients with healthy livers and patients with liver lesions. The quality of this method was found to be promising, with the AUC ROC for the testing set equal to 0.85 and sensitivity and specificity equal to 0.79. Further improvements to this pipeline and increasing the volume of the training set would likely further improve the quality of the results. The proposed system could aid practical radiological routines in the detection and differentiation of lesions of different types. We believe that further implementation of the proposed approach will improve the diagnostic process, for which the accurate recognition of lesions is mandatory but remains challenging.

## Figures and Tables

**Table 1:** MR protocol applied in standard abdominal imaging.

	TE	TR	FA	MX	VOX	FOV	CON	AV	PAT	DF	T
T2 (AX)	100	4000	180	182 × 320	1.3 × 1.3 × 4	200	4	1	2	30	1.38
T1 (AX)	2.58	215	70	154 × 256	1.6 × 1.6 × 4	400	4	1	2	30	0.44
DWI (AX)	71	7800	0	118 × 192	2.1 × 2.1 × 4	400	4	2,4,8	2	30	5.53

TE—time echo, TR—relaxation time, FA—flip angle, MX—imaging matrix, VOX—voxel size in mm^3^, FOV—field of view (mm), BW—bandwidth, AV—averages, CON—concentrations, TF–turbo factor, T—overall sequence time (minutes).

## Data Availability

Data can be made available on request by contacting the corresponding author.
